# The Effect of Posaconazole and Isavuconazole on the Pharmacokinetics of Erdafitinib in Beagle Dogs by UPLC-MS/MS

**DOI:** 10.3389/fphar.2021.749169

**Published:** 2021-11-29

**Authors:** Lan-hong Ruan, Ling-ling Fan, Kun Wang, Wan-qi Zhang, Xiao-jun Wang, Xiang-jun Qiu

**Affiliations:** ^1^ The First Affiliated Hospital, and College of Clinical Medicine of Henan University of Science and Technology, Luoyang, China; ^2^ School of Nursing of Henan University of Science and Technology, Luoyang, China; ^3^ School of Basic Medical Sciences, Henan University of Science and Technology, Luoyang, China

**Keywords:** posaconazole, isavuconazole, erdafitinib, UPLC-MS/MS, pharmacokinetic, beagle dog

## Abstract

**Objective:** A robust, quick, and reliable ultra-performance liquid chromatography–tandem mass spectrometry (UPLC-MS/MS) method for the quantification of erdafitinib in beagle dog plasma was developed and validated to evaluate the changes of posaconazole and isavuconazole on the pharmacokinetics of erdafitinib in beagle dogs, respectively.

**Methods:** This experiment adopted a three-period self-control experimental design. In the first period (group A), erdafitinib was orally administered to six beagle dogs at a dose of 4 mg/kg. In the second period (group B), the same six beagle dogs were orally given posaconazole at a dose of 7 mg/kg, and after 30 min, erdafitinib was orally given. In the third period (group C), isavuconazole at a dose of 7 mg/kg was given orally, and then, erdafitinib was orally given. At the different time points after erdafitinib was given in the three periods, the blood samples were collected. The concentration of erdafitinib was detected by the developed UPLC-MS/MS method. DAS 2.0 was used to calculate the pharmacokinetic parameters of erdafitinib.

**Results:** Erdafitinib had a good linear relationship in the range of 1–500 ng/ml, and the lower limit of quantification was 1 ng/ml. The precision, accuracy, extraction recovery, matrix effect, and stability meet the requirements of the guiding principles. After erdafitinib was combined with posaconazole, the C_max_ and AUC_0→t_ of erdafitinib increased by 27.19% and 47.62%, respectively, and the t_1/2_ was prolonged to 6.33 h. After erdafitinib was combined with isavuconazole, the C_max_ and AUC_0→t_ of erdafitinib increased by 23.13% and 54.46%, respectively, and the t_1/2_ was prolonged to 6.31 h.

**Conclusion:** A robust and reliable UPLC-MS/MS method was fully optimized and developed to detect the plasma concentration of erdafitinib in beagle dogs. Posaconazole and isaconazole could inhibit the metabolism of erdafitinib in beagle dogs and increase the plasma exposure of erdafitinib.

## Introduction

**FIGURE 1:**
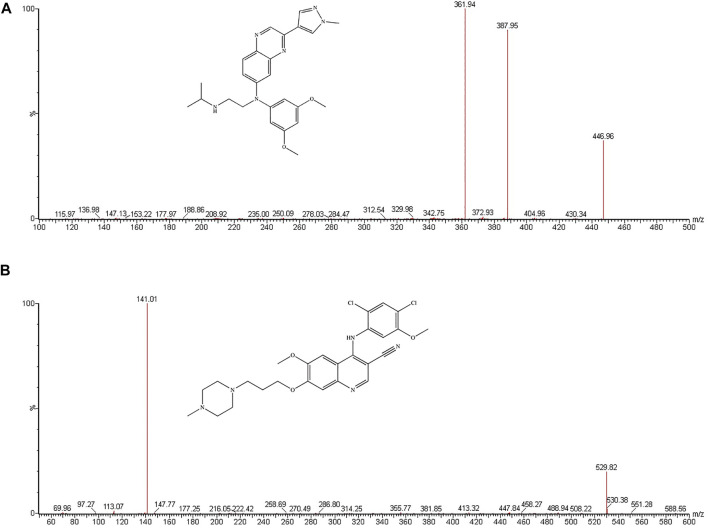
The chemical structure and mass spectra of erdafitinib **(A)** and bosutinib IS **(B)**.

Erdafitinib (JNJ-42756493, Figure 1A), an oral selective pan-fibroblast growth factor receptor (FGFR) inhibitor, inhibited enzymatic activity of FGFR1, FGFR2, FGFR3, and FGFR4. In cell lines expressing FGFR gene changes, including point mutations, amplifications, and fusions, erdafitinib inhibited FGFR phosphorylation and signal transduction and reduced cell viability. Meanwhile, erdafitinib showed antitumor activity in FGFR expressing cell lines and xenograft models derived from tumor types, such as bladder cancer (Roubal et al., 2020).

On April 12, 2019, erdafitinib had been granted accelerated approval for patients with locally advanced or metastatic urothelial carcinoma, with susceptible FGFR3 or FGFR2 genetic alterations, that had progressed during or after platinum-containing chemotherapy, including within 12 months of neoadjuvant or adjuvant platinum-containing chemotherapy by the Food and Drug Administration (FDA) (Food and Administration, 2019). The patients with metastatic or locally advanced, surgically unresectable, and urothelial carcinoma would potentially benefit from erdafitinib treatment (Wang et al., 2020c). In addition, erdafitinib was also being developed for the therapy of other solid tumors, including non–small cell lung cancer, liver cancer, cholangiocarcinoma, and pancreas cancer (Tabernero et al., 2015). Erdafitinib demonstrated a manageable safety profile and was well tolerated in Japanese patients with advanced or refractory solid tumors (Nishina et al., 2018).

Erdafitinib was mainly metabolized by CYP2C9 and CYP3A4 in the liver. CYP2C9 was estimated to be 39%, and CYP3A4 was estimated to be 20% of the total clearance of erdafitinib. There were no circulating metabolites in plasma, and the major drug-related moiety in plasma was unchanged erdafitinib (Roubal et al., 2020). Fluconazole or itraconazole or other moderate/strong CYP2C9 or CYP3A4 inhibitors might increase the plasma exposure of erdafitinib in healthy adults, and thus, the dose of drugs should be reduced when combined, or alternative combinations with no or minimal CYP2C9 or CYP3A4 inhibitory potential were used (Poggesi et al., 2020).

Patients with cancer, particularly solid tumors and hematologic malignancies, were at increased risk of invasive fungal diseases (IFDs). IFDs remained as the important causes of morbidity and mortality (Donnelly et al., 2020). A timely and sufficiently high exposure to the appropriate antifungal agent was crucial for IFDs (Bellmann and Smuszkiewicz, 2017). However, most of the patients with IFDs used antifungal drugs and other drugs, such as antitumor drugs, which often caused drug-drug interaction (DDI).

Posaconazole and isavuconazole were triazole antifungal drugs with a wide antimycotic spectrum and metabolized in the liver. Azoles were substrates and inhibitors of cytochrome P450 (CYP) isoenzymes and were therefore involved in numerous DDIs. Posaconazole was a strong inhibitor of CYP3A4 causing numerous DDIs, isavuconazole was a moderate CYP3A4 inhibitor, and numerous DDIs had to be considered (Bellmann and Smuszkiewicz, 2017).

Because the patients with cancer were often treated with multiple drugs, it would be necessary to explore whether erdafitinib could cause DDIs when combined with other drugs, such as posaconazole and isavuconazole. Consequently, it is necessary to invent and develop a method for the quantitative analysis of erdafitinib to evaluate the DDIs in pharmacokinetics. There were several literatures reported the pharmacokinetic profile of erdafitinib in patients using the LC-MS/MS methods (Nishina et al., 2018; Bahleda et al., 2019; Poggesi et al., 2020) and the HPLC-UV method (Elawady et al., 2021). On the basis of these reports, an ultra-performance liquid chromatography–tandem mass spectrometry (UPLC-MS/MS) method for detecting the concentration of erdafitinib in beagle dog plasma with bosutinib (Figure 1B) as internal standard (IS) was established and validated. In addition, the novel developed and validated UPLC-MS/MS method was successfully employed to investigate the pharmacokinetic DDI of erdafitinib wtih posaconazole and isavuconazole in beagle dogs.

## Materials and Methods

### Chemicals Materials

Erdafitinib and bosutinib (purity of >98%) were purchased from Beijing Sunflower and Technology Development Co., Ltd. (Beijing, China). Posaconazole (purity over 98%) was purchased from Jiangsu Aikang Biomedicine Research and Development Co., Ltd.; isavuconazole (purity over 98%) was purchased from Sigma (St Louis, MO, United States). Methanol and acetonitrile in this study were HPLC grade and were purchased from Tianjin Kermel Chemical Reagent Co., Ltd. (Tianjin, China). Analytically pure formic acid was purchased from Tianjin Kermel Chemical Reagent Co., Ltd. (Tianjin, China).

### Instruments

The ultra-high liquid chromatography instrument was Waters ACQUITY UPLC I-Class, including quaternary solvent manager, sample manager-flow through needle, and high-temperature column heater with active pre-heating (Waters, United States). The mass spectrometer was Waters XEVO TQD triple quadrupoles mass spectrometer, and the electro-spray ionization (ESI) source was used (Waters, United States). Other instruments included electronic analytical balance and vortex mixer, and ultra-pure water equipment.

### Animal Experiments

Six beagle dogs (male, weight 7.5 ∼ 9.5 kg) were purchased from Hubei Yizhicheng Biotechnology Co., Ltd. (Shiyan, Hubei). The animal license number was SCXK (Hubei) 2016–0020. The six beagle dogs were raised in the Laboratory Animal Center of Henan University of Science and Technology (Luoyang, China). All the experimental behaviors and operations were approved by the Institutional Ethics Committee of Henan University of Science and Technology (Luoyang, China). The experimental operation was carried out according to the Laboratory Animals Guidelines for Ethical Review of Welfare (GB/T 35,892–2018).

This experiment adopted a three-period self-control experimental design. All the beagle dogs fasted for 12 h before the experiment but were free to drink water. In the first period (group A), erdafitinib was dissolved in 0.5% carboxymethyl cellulose sodium solution and was orally administered to beagle dogs at a dose of 4 mg/kg. The blank blood was collected before erdafitinib was given, and then, at the different time points of 0, 0.5, 1, 1.5, 2, 3, 4, 6, 9, 12, 24, and 36 h, blood samples (about 1.0 ml) were collected from the veins of anterior and posterior limbs and taken into 1.5-ml heparinized EP tubes. The plasma were separated by centrifugation for 10 min at 10,000 rpm and frozen at −80°C for later analysis.

After a week of drug washout period, the second period (group B) of experiment was carried out. On the day of the experiment, the same six beagle dogs were orally given posaconazole at a dose of 7 mg/kg, and after 30 min, erdafitinib (4 mg/kg) was orally administered to beagle dogs. The time point of blood collection was the same as that of the first period.

Then, after a week of the second drug washout period, the third period (group C) of experiment was carried out. On the day of the experiment, the same six beagle dogs were orally given isavuconazole at a dose of 7 mg/kg, and after 30 min, erdafitinib was orally administered at a dose of 4 mg/kg. The time point of blood collection was the same as that of the first period.

### Solutions Preparation

Erdafitinib (10 mg) was accurately weighed and dissolved with methanol in a volumetric flask, and then, the volume was fixed with methanol to obtain a standard stock solution (1 mg/ml). IS stock solution (1 mg/ml) was prepared by the same method. The stock solution of erdafitinib (1 mg/ml) was diluted 10 times with methanol to obtain the standard solution of 100, 10, and 1 μg/ml for calibration curve and quality control (QC) samples. The stock solutions of erdafitinib and IS were stored in the refrigerator at −20°C.

The calibration curves with eight different concentration levels of erdafitinib were prepared by adding different concentration levels and volumes of standard application solution to blank beagle dog plasma, and the different concentration levels of calibration curve in beagle dog plasma were as follows: 1, 5, 10, 25, 50, 100, 250, and 500 ng/ml. QC samples of low, medium, and high concentration levels (2.5, 50, and 375 ng/ml) were prepared in the same way. The IS working solution at the concentration of 200 ng/ml was obtained by dilution of its stock solution with methanol.

### Plasma Sample Preparation

The beagle dog plasma (100 µl) to be tested was taken into 1.5-ml EP tube, and 20 µl of IS working solution was added and mixed well. In addition, 200 µl of 1 mol/L sodium hydroxide solution was added and mixed well. Then, 1 ml of ethyl acetate was added and vortexed for 1 min. The upper organic phase was transferred in another 1.5-ml EP tube and blow-dried with nitrogen flow. The residue was dissolved with 100 µl of mobile phase, take 50 µl into the sample of automatic injector, and 5 µl of supernatant was injected into UPLC-MS/MS for detection.

### Analytical Conditions

The chromatographic column was waters Acquity UPLC BEH C18 column (2.1 × 50 mm, 1.7 μm), the mobile phase was acetonitrile (A) and 0.1% formic acid in water (B), the gradient elution procedure was used, and the flow rate was 0.40 ml/min. The gradient elution procedure was as follows: 0–0.5 min A 10%, 0.5–1.0 min A 10%→90%, 1.0–2.0 min A 90%, and the termination time was 2 min. The volume of each injection was 5.0 µl, the column was set at 40°C, and the temperature of sample tray was set at 4°C.

In the positive ion mode, erdafitinib and IS were monitored by multiple reaction monitoring. The parent ion and daughter ion used for quantification were as follows: *m/z* 447.0 → 361.9 for erdafitinib and *m/z* 529.8 → 141.0 for IS, respectively. The cone voltage and collision energy of erdafitinib were 30 and 20 V, respectively, and the collision energy and cone voltage of IS were 25 and 20 V, respectively. The desolvation temperature was 1,000°C, and the capillary voltage was 2.0 kV. Masslynx 4.1 (Waters, United States) was used for data acquisition and instrument control.

## Method Validation

Methodology validation of UPLC-MS/MS included specificity, standard curve, lower limit of quantification (LLOQ), precision, recovery, matrix effect (ME), and stability. On the basis of the principles of the Industry Bioanalytical Method Validation proposed by FDA and the technical guidelines for non-clinical pharmacokinetics of chemical drugs, the UPLC-MS/MS method was validated (Food and Administration, 2018).

### Pharmacokinetics Study

Using batch processing method, the concentration levels of erdafitinib in groups A, B, and C were detected by the developed UPLC-MS/MS technique in this study. Then, DAS (Drug and Statistics, version 2.0) was used to calculate the important pharmacokinetic parameters of erdafitinib through the statistical moment method. The main pharmacokinetic parameters of erdafitinib were as follows: T_max_, C_max_, t_1/2_, CL, Vd, and AUC. Then, the mean concentration–time curve of erdafitinib was drawn.

### Statistical Analysis

The data were processed using SPSS 18.0 statistical software. Taking group A as the control group, the differences of pharmacokinetic parameters between group B and group A and between group C and group A were compared, respectively. The *p*-value was calculated by independent sample *t*-test, *p* < 0.05, which was statistically significant.

## Results

### Method Validation

### Selectivity

Six blank beagle dog plasma samples from different sources, standard solution of erdafitinib in blank beagle dog plasma, and samples after administration of erdafitinib were taken. According to the plasma sample processing method, the specificity of the UPLC-MS/MS method was investigated by comparing the chromatograms.

Under the above chromatographic and mass spectrometric conditions, erdafitinib and IS were separated completely, and the endogenous substances did not interfere with the detection. The representative chromatograms were shown in Supplementary Figure S2; A was the chromatogram of a blank beagle dog plasma sample, B was the chromatogram of plasma standard solution—a beagle dog plasma sample spiked with erdafitinib and IS, and C was the chromatogram of a beagle dog sample. The retention times of erdafitinib and IS were 0.92 and 0.81 min, respectively. The total running time for each sample was 2.0 min.

Chromatographic separation was based on the difference of adsorption and solubility of analytes by stationary phase. The components with strong adsorption or solubility had large partition coefficient and long retention time; on the contrary, the components with weak adsorption or solubility had small partition coefficient and short retention time. It could be seen from the chromatogram that the retention time of erdafitinib was slightly longer than that of IS. Therefore, under this chromatographic condition, the partition coefficient of erdafitinib was slightly larger than that of IS.

### Calibration Curve and LLOQ

After treatment with plasma sample treatment method, the plasma standard solutions of erdafitinib with the concentration levels of 1, 5, 10, 25, 50, 100, 250, and 500 ng/ml were prepared and detected. The peak areas of erdafitinib and IS were recorded, respectively. The concentration of erdafitinib was taken as the abscissa x, the ratio of the peak area of erdafitinib and IS was taken as the ordinate y, and the standard curve was drawn by the least square method with a weighted (1/*x* ^2^) to ensure the accuracy of low concentration point calculation. The lowest concentration of standard curve was the LLOQ.

At the concentration range of 1–500 ng/ml for erdafitinib, the typical regression equations of erdafitinib were *y* = 0.9009 *x + 0.3741 (*r* ^2^ = 0.999 3), which exhibited an excellent linearity. The LLOQ was 1.0 ng/ml, and the precision was below 8.67%, whereas the accuracy ranged from −2.33% to 4.29% (Table 1).

**TABLE 1:** Accuracy and precision of erdafitinib in beagle dog plasma (*n* = 6).

Added (ng/ml)	Intra-day			Inter-day		
	Found (ng/ml)	RSD%	RE%	Found (ng/ml)	RSD%	RE%
1	0.98 ± 0.08	8.67	−2.33	1.04 ± 0.08	8.13	4.29
2.5	2.54 ± 0.23	8.93	1.41	2.56 ± 0.19	7.40	2.45
50	50.16 ± 3.66	7.31	0.21	48.46 ± 2.99	6.18	−3.08
375	369.84 ± 14.29	3.86	−1.37	376.99 ± 18.27	4.85	0.53

Recovery and matrix effect.

At low, medium, and high concentration levels (2.5, 50, and 375 ng/ml), the precision and accuracy were estimated by sextuple detection of QC samples over three consecutive days. The precision was expressed by relative standard deviation (RSD, %), and the accuracy was expressed by relative error (RE, %).

Table 1 showed the results of intra-day and inter-day precision and accuracy of erdafitinib. The precision (% RSD) did not exceed 8.93%. Accuracy (% RE) was in the range from −3.08% to 4.29% at low, medium, and high concentration levels (2.5, 50, and 375 ng/ml).

At low, medium, and high concentration levels (2.5, 50, and 375 ng/ml), by comparing the ratio of the peak area of erdafitinib before and after the extraction, the extraction recovery was calculated. ME was also calculated in six replicates by comparing the response of erdafitinib in plasma matrix after extraction with that in neat solution, and six batches of blank matrix were obtained from blank plasma of different beagle dogs.

At low, medium, and high concentration levels (2.5, 50, and 375 ng/ml), the extraction recovery of erdafitinib was within the range of 79.22% ∼ 82.25% (Table 2), and the ME values for erdafitinib were 98.71% ∼ 102.53% (Table 2). ME did not affect the detection of samples.

**TABLE 2:** Results of recovery and matrix effect of erdafitinib (*n* = 6).

Added (ng/ml)	Recovery (%)		Matrix effect (%)	
	Means ± SD	RSD (%)	Means ± SD	RSD (%)
2.5	79.22 ± 3.20	4.04	102.53 ± 4.95	4.83
50	82.25 ± 4.14	5.03	98.71 ± 5.75	5.83
375	81.77 ± 3.50	4.27	101.61 ± 3.24	3.19

### Stability

At low, medium, and high concentration levels (2.5, 50, and 375 ng/ml), the stability of plasma samples was investigated under four different storage conditions: processed samples at 4°C in auto-sampler tray for 6 h, room temperature for 3 h, −80°C for 4 weeks, and three freeze-thaw cycles (−80°C to 25°C).

All results of the stability were summarized in Table 3, and it was found that erdafitinib was stable under the conditions described above.

**TABLE 3:** Stability of erdafitinib in beagle dog plasma under different storage conditions (*n* = 6, each concentration).

Added (ng/ml)	Autosampler 4°C, 6 h		Room temperature, 3 h		−80°C, 4 weeks		Three freeze-thaw	
	RSD (%)	RE (%)	RSD (%)	RE (%)	RSD (%)	RE (%)	RSD (%)	RE (%)
2.5	9.43	0.18	7.10	−2.45	8.05	−1.69	6.84	0.33
50	5.99	−1.34	8.01	3.08	5.82	−4.43	6.14	−3.37
375	4.00	2.13	4.62	1.42	2.87	−1.29	3.96	−0.61

### Ruggedness

At similar operational and environmental conditions, the plasma standard solutions of erdafitinib with the concentration level of 250 ng/ml were prepared and analyzed by two different analysts and repeated six times, respectively. The peak area of erdafitinib was recorded and RSD was calculated. The method was found to be rugged (RSD, 2.84%).

The stock solution stability of erdafitinib and IS was assessed individually after 6 weeks of storage at −20°C, respectively. This was done by comparing the signal response for six replicates of erdafitinib or IS (10 μg/ml) made using the freshly prepared stock solution with the signal response for six replicates of erdafitinib or IS (10 μg/ml) made using the stock solution prepared 6 weeks earlier.

Stock solution stability was tested after 6 weeks for erdafitinib and IS, and it was found that the stock solution of erdafitinib and IS was stable for up to 6 weeks.

### Pharmacokinetics Study

**FIGURE 2:**
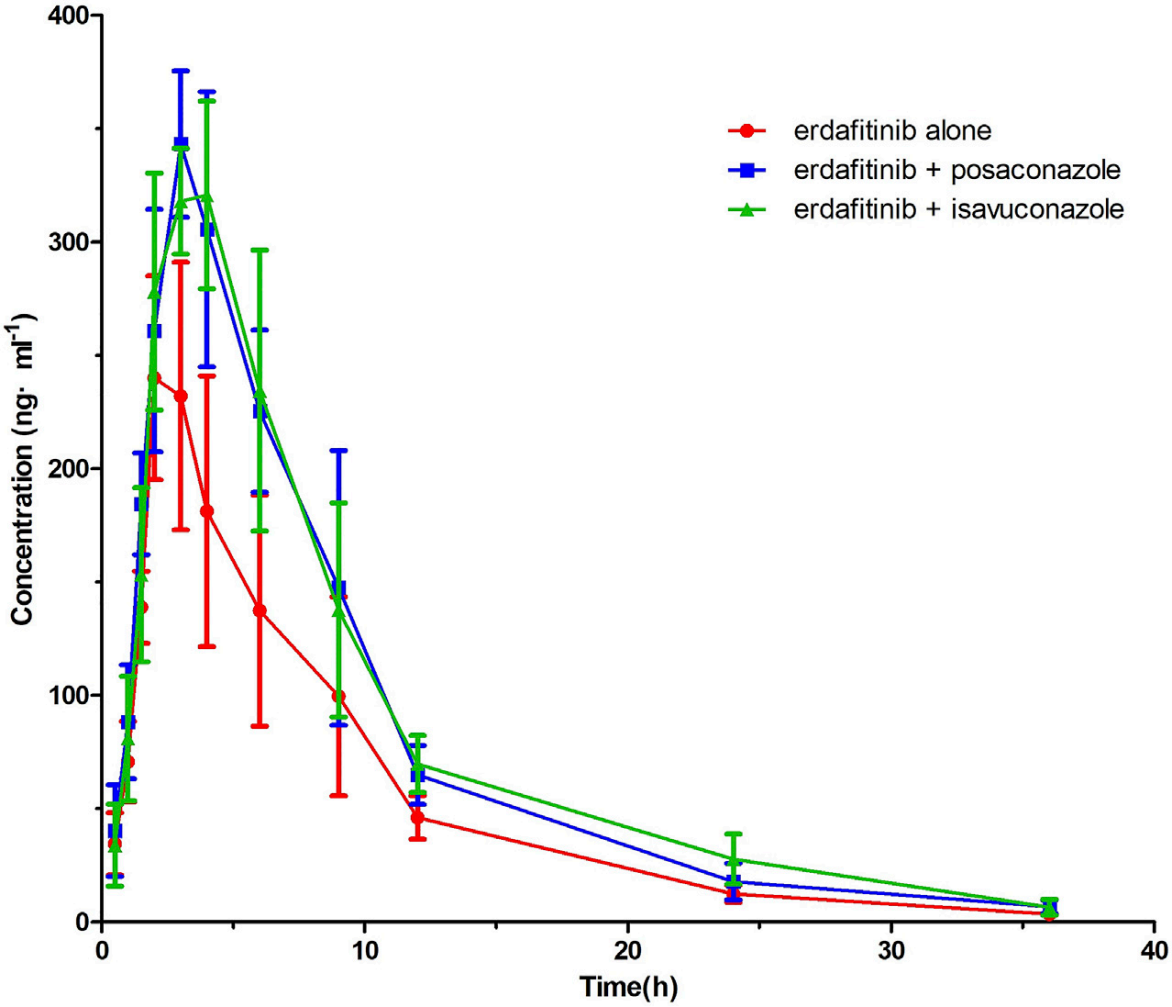
Mean plasma concentration–time profile of erdafitinib after administration to six beagle dogs dosed alone and incombination with posaconazole or isavuconazole.

Figure 2A displayed the average plasma drug concentration–time curves of erdafitinib in groups A and B, and the average plasma concentration–time curves of erdafitinib in groups A and C were displayed in Figure 2B. The main parameters of pharmacokinetic of erdafitinib in groups A, B, and C were summarized in Table 4.

**TABLE 4:** Pharmacokinetic parameters of erdafitinib after oral dose to beagle dogs (*n* = 6, Means ± SD).

Parameters	Group A	Group B	Group C
T_max_ (h)	2.67 ± 0.82	3.33 ± 0.52	3.17 ± 0.75
C_max_ (ng/ml)	282.12 ± 25.28	358.84 ± 30.59*	347.38 ± 27.21*
t_1/2_ (h)	5.96 ± 1.56	6.30 ± 1.92	6.32 ± 1.85
CLz/F (L/h/kg)	2.06 ± 0.38	1.38 ± 0.23*	1.30 ± 0.17*
Vz/F (L/kg)	18.17 ± 7.78	12.43 ± 3.93*	11.84 ± 3.44*
AUC_0→t_ (ng/mL•h)	1966.98 ± 394.36	2,903.57 ± 490.64*	3,038.13 ± 409.64*
AUC_0→∞_ (ng/mL•h)	1999.33 ± 379.87	2,961.66 ± 527.27*	3,114.28 ± 425.21*

Note: group A, erdafitinib alone (4 mg/kg); group B, erdafitinib (4 mg/kg) + posaconazole (7 mg/kg); group C, erdafitinib (4 mg/kg) + isavuconazole (7 mg/kg).

*Compared with group A, the difference was statistically significant (*p* < 0.05).

The results showed that, after erdafitinib was combined with posaconazole, the C_max_ and AUC_0→t_ of erdafitinib increased by 27.19% and 47.62%, respectively. After erdafitinib was combined with isavuconazole, the C_max_ and AUC_0→t_ of erdafitinib increased by 23.13 %and 54.46%, respectively.

## Discussion

### Method Validation and Improvement

UPLC-MS/MS has the advantages of high sensitivity, strong specificity, short analysis time, and good reproducibility. Therefore, it is often used in the detection of biological samples and the study of pharmacokinetics and DDI (Wang et al., 2020b; Yang et al., 2021).

In this study, we explored ESI positive and negative to find the most sensitive ionization modes of erdafitinib and IS. It demonstrated that positive ion mode exhibited higher mass response than negative ion mode. As indicated in Figure 1, erdafitinib and IS showed protonated molecular ion [M + H]^+^ at *m/z* 447.0 and 529.8, respectively, and the most abundant product ions were *m/z* 361.9 and 141.0, respectively. As a result, the parent ion–to–daughter ion ratio for erdafitinib was *m/z* 447.0 → 361.9 and for IS was *m/z* 529.8 → 141.0.

The mobile phase should meet the requirements of HPLC and LC/MS. Volatile salts should be added to the mobile phase as far as possible, and surfactants and other non-volatile buffers should not be used as far as possible, such as phosphoric acid buffer. Phosphate and other non-volatile buffer salts will precipitate in the ion source and plug the capillary. In this experiment, formic acid, water, and ammonium formate were first selected to investigate the influence of mobile phase on peak pattern and peak intensity. Finally, 0.1% formic acid and acetonitrile were used as mobile phase, which could increase the ionization degree of the sample, increase the signal intensity and improve the peak pattern. The mobile phase gradient elution method could complete a detection in 2 min. The detection was faster than that reported (Elawady et al., 2020).

In the field of pharmaceutical analysis, sample pretreatment and purification was an important step. Improving the pretreatment method could not only protect the detection instrument from pollution and prolong its service life but also reduce the detection matrix and improve the sensitivity and selectivity. It was reported that the plasma was pretreated by solid phase extraction (SPE) in the process of determination of erdafitinib in the human plasma with LC-MS/MS (Elawady et al., 2020). Because SPE needed special extraction column, the treatment process was relatively cumbersome. In this study, through the exploration of a series of pretreatment methods, the ethyl acetate liquid-liquid extraction method was selected to pretreat the plasma samples, and the sodium hydroxide solution was added to adjust the pH value of the samples, which could significantly improve the extraction recovery rate of erdafitinib. The influence of matrix interference on the determination was reduced.

### Drug-Drug Interaction

DDI refers to the phenomenon that one drug changes the pharmacological effect of the other drug when taking more than two drugs at the same time or successively. Pharmacokinetic DDIs are those that affect the absorption, distribution, metabolism, and excretion of a concomitantly administered drug (Rabba et al., 2020). This change can lead to the change of the concentration of drug at the action site, thus affecting the duration and magnitude of the effect (Vázquez et al., 2020).

DDIs are one of the important factors leading to adverse reactions and are often associated with toxicity or therapeutic failure (Wang et al., 2020a), but sometimes, DDIs are beneficial to patients thought improving the bioavailability of drugs and producing synergistic or additive effects (Gerber et al., 2018). In any case, clinicians must be familiar with DDIs to improve prescribing tools.

Erdafitinib was a potent oral pan-FGFR inhibitor being developed as oncology drug for patients with alterations in the FGFR pathway. The geometric mean C_max_ of erdafitinib was 136 ng/ml after a single dose of erdafitinib in healthy adults (Poggesi et al., 2020). Patients with advanced or refractory solid tumors after treatment with different doses of erdafitinib, the exposure of erdafitinib increased in a dose-dependent manner, the median T_max_ ranged from 2–3 h after the initial dose to 2–6 h after multiple daily dosing (Nishina et al., 2018). After mice were given erdafitinib (30 mg/kg), C_max_ was about 810 ng/ml and t_1/2_ was about 1.73 h, and after mice were given erdafitinib (10 mg/kg), C_max_ was about 110 ng/ml and t_1/2_ was about 2.36 h (Elawady et al., 2021). The PK results of this study showed that, after beagle dogs were given erdafitinib (4 mg/kg), C_max_ was about 282 ng/ml, which was close to human C_max_ when converted according to dose (Poggesi et al., 2020).

Our previous research results show that posaconazole significantly increased the concentration of selinexor (a selective nuclear export inhibition) in rats, fluconazole, itraconazole, and isavuconazole that have significant effects on pharmacokinetics of selinexor and increased plasma exposure of selinexor in rats (Li et al., 2020; Zhou et al., 2021).

The results of the study showed that, after erdafitinib was combined with posaconazole, the C_max_ and AUC_0→t_ of erdafitinib increased by 27.19 and 47.62%, respectively, and the t_1/2_ was prolonged to 6.33 h; after erdafitinib was combined with isavuconazole, the C_max_ and AUC_0→t_ of erdafitinib increased by 23.13 and 54.46%, respectively, and the t_1/2_ was prolonged to 6.31 h. It is suggested that posaconazole and isavuconazole could affect the pharmacokinetics of erdafitinib in beagle dogs and increase the plasma exposure of erdafitinib. Because posaconazole was a strong inhibitor of CYP3A4, isaconazole was a moderate inhibitor of CYP3A4, so posaconazole and isaconazole could slow down the metabolism of erdafitinib by inhibiting CYP3A4. Therefore, when erdafitinib was combined with posaconazole and isavuconazole, possible pharmacokinetics DDIs should be considered. However, after all, human drug metabolism enzymes were different from beagle dogs, so the experimental results could only be used as a theoretical reference. DDIs in humans would need further validation. Therefore, the results of this study still had some limitations.

## Conclusion

A robust, quick, and reliable UPLC-MS/MS method was fully optimized and developed to detect the concentration of erdafitinib in beagle dogs. The method had been successfully applied to the pharmacokinetic DDI study of erdafitinib in beagle dogs. Posaconazole and isavuconazole could inhibit the metabolism of erdafitinib in beagle dogs and increase the plasma exposure of erdafitinib. Therefore, when erdafitinib was combined with posaconazole and isavuconazole, the potential pharmacokinetic DDIs should be considered.

## Data Availability

The original contributions presented in the study are included in the article/Supplementary Material; further inquiries can be directed to the corresponding author.
