# Supplementary figures and images for: The Effect of Posaconazole and Isavuconazole on the Pharmacokinetics of Erdafitinib in Beagle Dogs by UPLC-MS/MS
Source: Front Pharmacol. 2021 Nov 29;12:749169. doi: 10.3389/fphar.2021.749169 (PMC8666568; doi:10.3389/fphar.2021.749169)

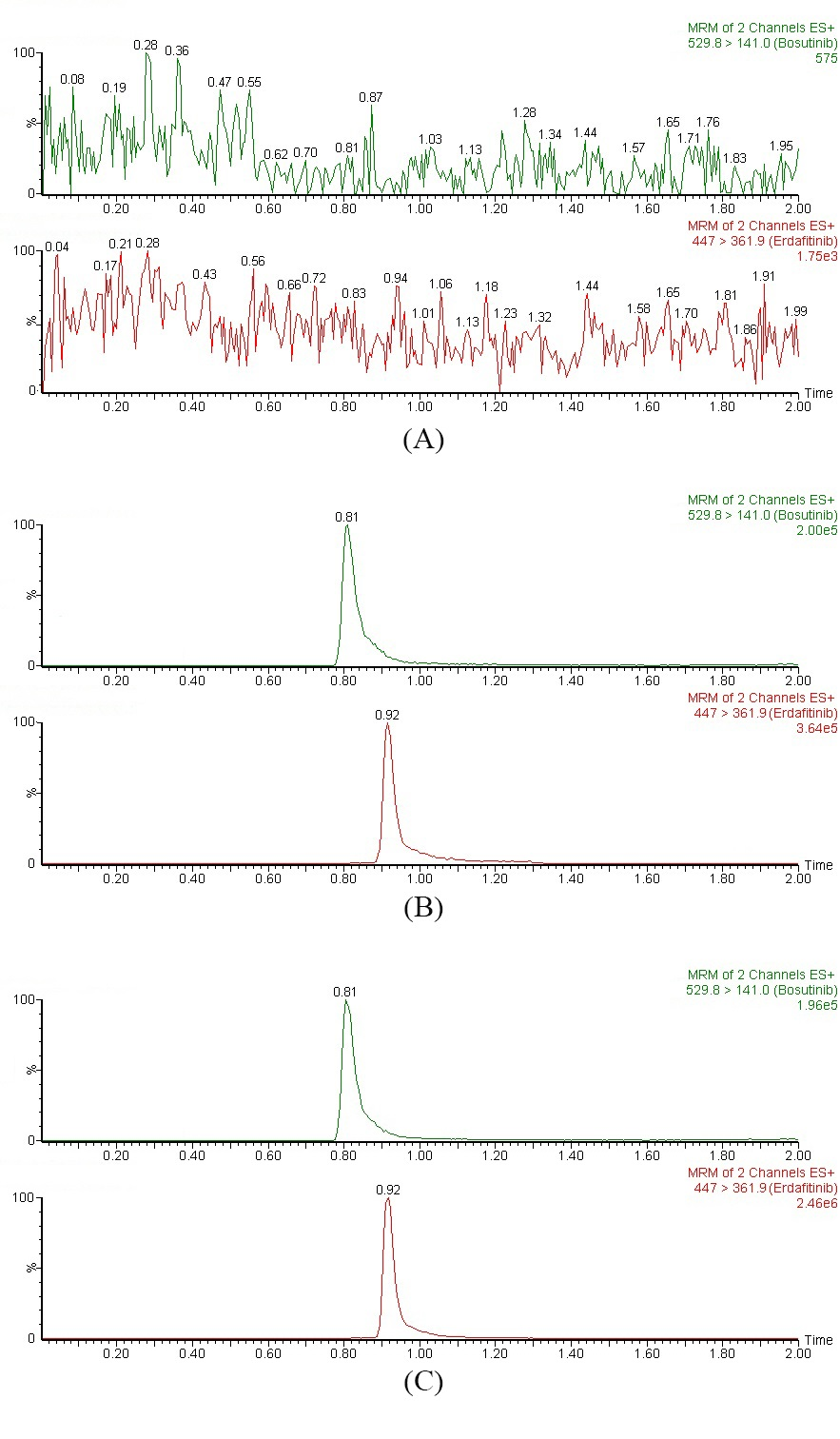

Supplement: Supplementary file 1 [file Image1.TIF]
